# Supplementary material for: Predicting visual function by interpreting a neuronal wiring diagram
Source: Nature. 2024 Oct 2;634(8032):113–23. doi: 10.1038/s41586-024-07953-5 (PMC11446822; doi:10.1038/s41586-024-07953-5)
Supplement: Supplementary file 6 — CRF and ERF predictions for individual TmY4 and TmY9 cells. Analogous to Supplementary Data 3, but for TmY target types. Shown are the top four monosynaptic pathways, the strongest pathway passing through each of the top ten intermediary types (ranking from Extended Data Fig. 7), and the trisynaptic pathway Tm1–TmY–Dm3–TmY (see the section entitled Prediction of spatial normalization). [file 41586_2024_7953_MOESM6_ESM.zip › DataS4/TmY9qΓèÑ/720575940620524747.pdf]

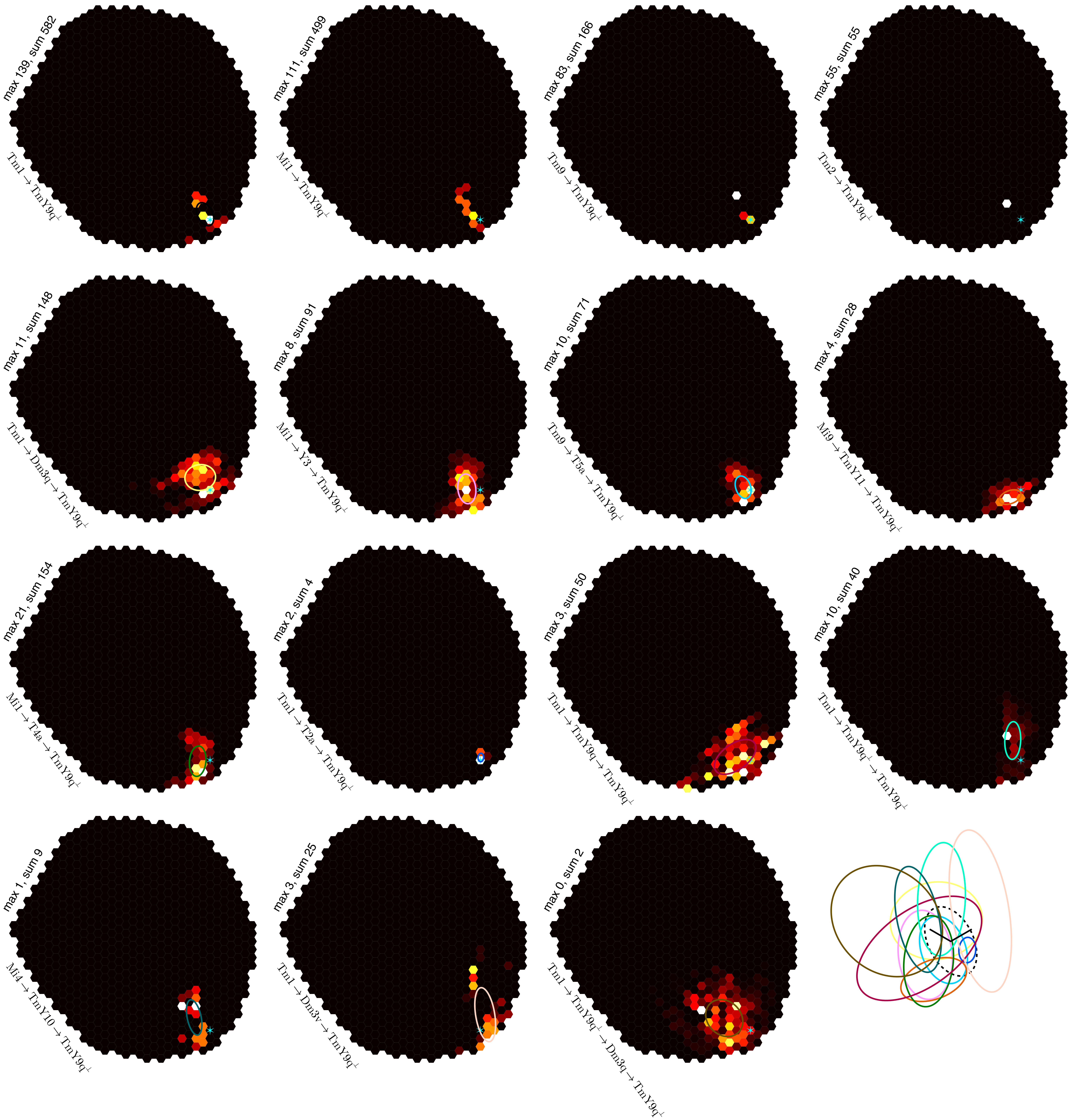

max 139, sum 582
Tm1 → TmY9q⊥
max 111, sum 499
Mi1 → TmY9q⊥
max 83, sum 166
Tm9 → TmY9q⊥
max 55, sum 55
Tm2 → TmY9q⊥
max 11, sum 148
Tm1 → Dm3q → TmY9q⊥
max 8, sum 91
Mi1 → Y3 → TmY9q⊥
max 10, sum 71
Tm9 → T5a → TmY9q⊥
max 4, sum 28
Mi9 → TmY11 → TmY9q⊥
max 21, sum 154
Mi1 → T4a → TmY9q⊥
max 2, sum 4
Tm1 → T2a → TmY9q⊥
max 3, sum 50
Tm1 → TmY9q → TmY9q⊥
max 10, sum 40
Tm1 → TmY9q⊥ → TmY9q⊥
max 1, sum 9
Mi4 → TmY10 → TmY9q⊥
max 3, sum 25
Tm1 → Dm3v → TmY9q⊥
max 0, sum 2
Tm1 → TmY9q⊥ → Dm3q → TmY9q⊥
